# Emotional Health of People with Visual Impairment Caused by Retinitis Pigmentosa

**DOI:** 10.1371/journal.pone.0145866

**Published:** 2015-12-29

**Authors:** Keziah Latham, Mohammad Baranian, Matthew Timmis, Shahina Pardhan

**Affiliations:** 1 Visual Function and Physiology Research Group, Anglia Ruskin University, Cambridge, United Kingdom; 2 Vision and Eye Research Unit, Anglia Ruskin University, Cambridge, United Kingdom; 3 Sport and Exercise Science Research Group, Anglia Ruskin University, Cambridge, United Kingdom; Justus-Liebig-University Giessen, GERMANY

## Abstract

**Purpose:**

To understand the emotional difficulties associated with living with the ocular condition Retinitis Pigmentosa, and to examine the functioning of a self-report instrument used to assess this construct.

**Methods:**

The difficulty of goals and tasks in the emotional health domain of the Dutch ICF Activity Inventory were rated by 166 people with Retinitis Pigmentosa in a cross-sectional study. Demographic factors were also assessed.

**Results:**

Responses to the 23 emotional health tasks were Rasch analysed and could be used to form either one 20 item overview scale with some multidimensionality, or three unidimensional subscales addressing feelings (4 items), communicating visual loss (5 items) and fatigue (7 items). The most difficult individual tasks related to communicating visual loss to other people, and dealing with feelings such as frustration, anxiety and stress. The use of mobility aids and female gender were associated with increased difficulty with emotional health, explaining 19% of the variance in the overview scale.

**Conclusions:**

The emotional health domain of the Dutch ICF Activity Inventory is a valid tool to assess emotional difficulties arising from visual loss. Interventions to aid people with Retinitis Pigmentosa deal with emotional difficulties should particularly address communicating vision loss effectively to others and coping with negative feelings.

## Introduction

Retinitis Pigmentosa (RP) is an inherited condition with several genotypes, causing bilateral retinal dystrophy [1]. Such dystrophies are the commonest cause of registerable visual impairment in people of working age in the United Kingdom (UK) [2]. The visual impairments associated with the initial stages of RP typically include impaired scotopic vision and reduced peripheral visual field [3]. In later stages, central vision can become affected with reduced visual acuity, contrast sensitivity and colour vision [3]. These impairments lead to particular difficulty with mobility [4], and as the condition affects people from a young age [5] also has a significant impact on working activities [4].

Acquired visual loss has the potential to have a profound impact on an individual’s mental health and emotional well-being. It is known that older people who experience sight loss have higher rates of depression than sighted peers [6–8], and the depression can persist for significant periods of time [9, 10]. In its most extreme effects, older people with visual impairment have higher mortality rates [11, 12] and may be more likely to commit suicide [13–15] than those with good vision. However, effective emotional adjustment to experiencing visual loss is possible [16, 17], and is associated with greater acceptance of visual loss, better social support, and a positive attitude [16, 17].

However, much of the research examining emotional health in acquired visual loss has considered people with age-related macular degeneration (AMD) [18] or older people in general [19]. As RP presents at younger ages, and affects vision differently to AMD by predominantly affecting peripheral rather than central visual function, it is of value to independently assess the impact of this particular condition on people’s emotional health.

Questionnaires have been developed to examine the effects of visual loss on daily living [20]. Of these instruments, the importance of emotional health as an area requiring potential rehabilitation for those with visual impairment has been noted in the construction of the Dutch ICF Activity Inventory (D-AI; [21]). The D-AI is an instrument for assessing the rehabilitative needs and priorities of visually impaired people, and is used on a routine basis in the Netherlands [22]. It was designed using the framework of the International Classification of Functioning, Disability and Health [23], which specifies nine ‘Activity and Participation’ domains (learning and applying knowledge, general tasks and demands, communication, mobility, self care, domestic life, interpersonal interactions and relationships, major life areas, and community, social and civic life). The additional domain of ‘emotional health’ was added to the D-AI on the basis of its importance in focus group responses from both patients and visual rehabilitation professionals [24] and has a high priority in rehabilitation needs, exceeded in importance only by goals of ‘learning and applying knowledge’ such as reading [25].

The D-AI is administered by firstly asking people to rate the importance and difficulty of 47 goals that underlie the ten domains of the instrument. Following this, the difficulty of tasks underlying the most important and difficult goals can be assessed in order to develop a rehabilitation plan for an individual [22], or to provide a more detailed understanding of the issues causing difficulty with specific goals. It has been shown that use of the D-AI to identify rehabilitation needs in a structured way identifies far more needs (on average 24 vs. 6.7 rehabilitation needs) than when assessed by a usual case history method [22].

The D-AI has been analysed at goal level using Rasch analysis to validate and evaluate the questionnaire [4]. Rasch analysis is a probabilistic measurement model used to construct a linear measure from ordinal observations [26], allowing both application of parametric statistics to responses and detailed evaluation of questionnaire performance [27], such as the extent to which questions address the same issue or construct (unidimensionality), the ordering of the items in terms of difficulty, and the ordering of respondents in terms of ability. In a group of people with visual impairment due to Retinitis Pigmentosa [4], responses to the emotional health goals of the D-AI were not consistent with the remainder of the goals related to specific visual activities of daily living, and were therefore removed from the instrument as they did not support its unidimensionality [27]. However, in provisional ordinal analysis of the data [28], the emotional health goals of the instrument were the area of most difficulty for those in the early stages of visual loss, who were not registerable as visually impaired. Therefore, it is of interest to examine responses to the emotional health questions of the D-AI in more detail at task level, separated from other areas of the instrument, in order to understand specific areas of difficulty in maintaining emotional health in the face of vision loss. The emotional health domain of the D-AI has previously been evaluated in a sample of people with mixed causes of low vision, but using classical analysis techniques [29]. It was found that the most appropriate construct for the emotional health tasks was to underpin two separate goals of ‘emotional health’ and ‘fatigue’.

The aim of this study was therefore to assess the difficulty of tasks associated with emotional health for those with visual loss due to Retinitis Pigmentosa. Understanding the aspects causing most difficulty could provide evidence to guide the development of appropriate support programs. Integral to this aim is an examination of the performance of the emotional health domain of the D-AI using Rasch analysis in this sample, extending the validation of the instrument beyond that already available [4, 25].

## Methods

### Participants

Participants were recruited by advertising the study through the charity Retinitis Pigmentosa Fighting Blindness (RPFB), and by contacting people with RP who had taken part in a previous study [4] and had agreed that they could be contacted about future studies. Note that a different sample of people participated in the present study as compared to previous work on the D-AI at goal level only [4]. Potential participants were provided with the internet address of the online questionnaire, and contact details of the researchers for further information. Inclusion criteria were that the participant self-reported that they had RP, and were 18 years of age or over. Ethical approval was received from Anglia Ruskin University Faculty of Science and Technology Research Ethics Committee (DREP/N0514.3), and the tenets of the Declaration of Helsinki were upheld. Once the nature of the study had been explained, participants gave their informed consent to take part by checking a box on the web page. Participants could not proceed to the questionnaire without giving their informed consent.

### Procedures

Information regarding the participants’ age, gender, duration of visual impairment, use of mobility aids, and visual impairment registration status were requested. In the UK, people can be registered as sight impaired (SI) with full visual field and visual acuity (VA) 3/60–6/60, VA up to 6/24 with a ‘moderately contracted’ visual field, or VA 6/18 or better if there is a ‘gross’ field defect. Severely sight impaired (SSI) registration is available to those with VA <3/60 and full visual field, VA between 3/60 and 6/60 with a ‘significantly contracted’ field of vision, or VA of >6/60 with a ‘severely contracted’ field of vision [30]. It should be noted however, that interpretation of these guidelines with respect to field loss is not consistent [31].

Participants were asked to rate the importance and difficulty of the two goals of the Dutch ICF Activity Inventory (D-AI) underpinning the emotional health subscale [32]. These are: ‘Is maintaining your emotional health and accepting your visual impairment difficult for you to achieve due to your visual impairment?’ and ‘Do you have difficulties with fatigue, concentration and balancing energy levels due to your visual impairment?’ Goals were scored according to the following scale: 0 = not important or not applicable; 5 = no difficulty; 4 = slight difficulty; 3 = moderate difficulty, 2 = severe difficulty, 1 = impossible without help.

Participants who responded that a goal was relevant and of some difficulty (i.e. scores of 4–1) were asked how difficult a range of tasks underpinning this goal were (outlined in Tables 1 and 2). Response options were: 0 = not applicable (considered as missing data); 5 = no difficulty; 4 = slight difficulty; 3 = moderate difficulty, 2 = severe difficulty, 1 = impossible without help. All questions were administered in English.

**Table 1:** Task questions underlying the goal ‘maintaining emotional health’.

Item no	Task
1	Deal with feelings of loneliness
2	Deal with gloomy or sad feelings
3	Deal with frustration, anger or despair
4	Deal with feelings of anxiety
5	Deal with stress
6	Deal with feelings of inferiority
7	Enjoy shared / group activities
8	Be open about your visual impairment with strangers
9	Be open about your visual impairment with acquaintances
10	Deal with misunderstandings from others because of your visual impairment
11	Explain to others what you can and cannot see
12	Ask for help from people you know
13	Refuse help from people you know
14	Deal with changing roles and relationships because of your visual impairment (with people close to you)

Participants were asked: ‘These questions relate to your emotional health and accepting your visual impairment. How difficult are each of the tasks below to do without the assistance of another person, but with any assistive devices that you use?’

**Table 2:** Task questions underlying the goal ‘fatigue’.

Item no	Task
15	Sustain your daily activities during the day, such as shopping, cooking or arranging things
16	Finish your daily activities in time
17	Get somewhere without getting too tired
18	Stay focused and concentrated
19	Perform your daily activities without suffering from discomfort in the eyes (e.g. eye strain)
20	Perform daily activities without suffering from other symptoms (such as neck, back or headache)
21	Balance your energy during the day (e.g. so that you have some energy left at the end of the day)
22	Do things in your spare time (such as hobbies or social contacts)
23	Keep a day and night rhythm

Participants were asked: ‘These questions relate to coping with fatigue and balancing energy levels. How difficult are each of the tasks below to do without the assistance of another person, but with any assistive devices that you use?’

### Analysis

Rasch analysis of the task responses was undertaken using Winsteps version 3.80.1 [33]. Person and item measures are produced in logits, or log odds units, which represent the likelihood of a person having the ability to achieve an item, or an item being achievable for a person. The average logit value for both persons and items is arbitrarily set to zero. The ordinal scale of difficulty from 1 to 5 with higher numbers indicating less difficulty differed from the original D-AI, where higher scores indicated greater difficulty. The scale used was chosen so that on the resulting interval scale, a higher positive person measure indicates that an individual perceives that they have greater ability with the items, and a higher item measure indicates that an individual would need greater ability in order to achieve the task, therefore representing a ‘harder’ item. Note that with Rasch analysed data, missing data from questions that are not applicable to individuals do not affect the legitimacy of the scores obtained.

Rasch analysis was undertaken with a single Andrich rating scale model [34]. Initially, category thresholds were examined to determine if all categories were utilised, that categories were used in order of functional ability, and that each category was the most probable response at some point on the ability scale. Note that as the location of category thresholds differs between scales, the person and item logit values derived in analyses can be only be compared within scales, and not between scales. The fit of items to a unidimensional construct was assessed, with acceptable fit defined as infit and outfit meansquare (mnsq) values within a range of 0.6 to 1.4 [4, 35]. Any ill-fitting goals were removed iteratively, with the most misfitting removed first and the analysis repeated until all item fits were within the specified range.

The reliability indices of the resulting instrument were assessed in terms of person separation statistics, which provide an indication of the instrument’s ability to discriminate between respondents: person separation and person reliability should be greater than the suggested minima of 2.0 and 0.80 respectively [36]. Further, item separation statistics provide an indication of how reliably ordered the items are in terms of difficulty: item separation and item reliability should be in excess of suggested minima of 3.0 and 0.90 [36]. Targeting, or the difference between mean item and person measures, should ideally be less than 1.0 logit [37].

Uniform differential item functioning (DIF), or item bias, was examined to determine whether responses to any of the items varied significantly depending on the type of person responding to the questionnaire as defined by the demographic variables assessed. DIF tests the assumption that an item has extra difficulty for all those in one classification group. To be noticeable, the difference in difficulty of an item between two groups should have a DIF contrast of at least 0.5 logits [38] with a statistically significant probability (p<.01) indicating little likelihood of this difference occurring by chance [38]. DIF greater than 1.0 logit may damage the integrity of the scale and merit action in terms of splitting the item or removing it.

Further assessment of the unidimensionality of the instrument is important to demonstrate the extent to which an instrument assesses a single latent trait. In Rasch residual-based principal components analysis (PCA), the variance in the data that is accounted for by the Rasch dimension is first considered, with at least 60% of variance explained by the primary measure considered to demonstrate reasonable overall unidimensionality [37, 39] in the instrument. The unexplained variance, or residuals, are then decomposed to look for patterns that may indicate a secondary dimension to the data rather than random noise. For potential additional dimensions to be considered, the contrast found within the residuals after the primary model has been extracted has to have at least the strength of two items, i.e., an eigenvalue of at least 2.0, because this is close to that seen within random data [40].

Comparisons between parameters were assessed using SPSS version 20 (IBM, New York, USA). Relationships between two groups were determined using independent samples t-tests, and one factor ANOVA was used for registration status data where there were three groups. For comparison between two continuous variables, Pearson’s r was determined.

## Results

One hundred and sixty six people completed the online questionnaire. There were 91 female and 75 male participants, with a mean age of 50±16 years (range 18–83 years), who reported having been visually impaired for a mean duration of 22±16 years (range 6 months-70 years). Men and women were of a similar age (t(163) = 0.88, p = 0.38), but men reported a longer duration of visual impairment than women (males: 25.5±17.3 years, females 18.6±14.0 years, t(163) = 2.85, p = .005). Seventeen people were not registered as visually impaired, 63 were registered as sight impaired (SI) and 86 were registered as severely sight impaired (SSI), and registration status did not differ by gender (F(1,164) = 0.79, p = .38). Eighty two people used mobility aids (cane and / or guide dog) and 84 did not. Use of mobility aids did not differ by gender (t(164) = -0.95, p = .35), but mobility aid users were older (users: 54.5±14.5 years, non-users 45.4±15.9 years, t(163) = 3.86, p = .000), had been visually impaired for longer (users: 25.4±17.5 years, non-users 18.0±13.2 years, t(163) = 3.86, p = .000), and were more severely visually impaired as indicated by their registration status (F(1,164) = 50.4, p = .000).

Of the total number of 166 respondents, 149 reported that at least one of the two emotional health goals was relevant and of some difficulty to them, and were asked the task questions. Each task question was relevant to between 116 and 135 people (70–81% of total respondents). When all task items (1–23; Tables 1 and 2) underlying the domain of emotional health were considered together, not all items fell within the fit range of 0.6–1.4, indicating deviation from the underlying unidimensional construct of emotional health. Items 13, 8 and 9 were removed due to underfit, with fit values up to 1.78 in the initial analysis, after which all items fell within the defined fit limits.

**Fig 1:**
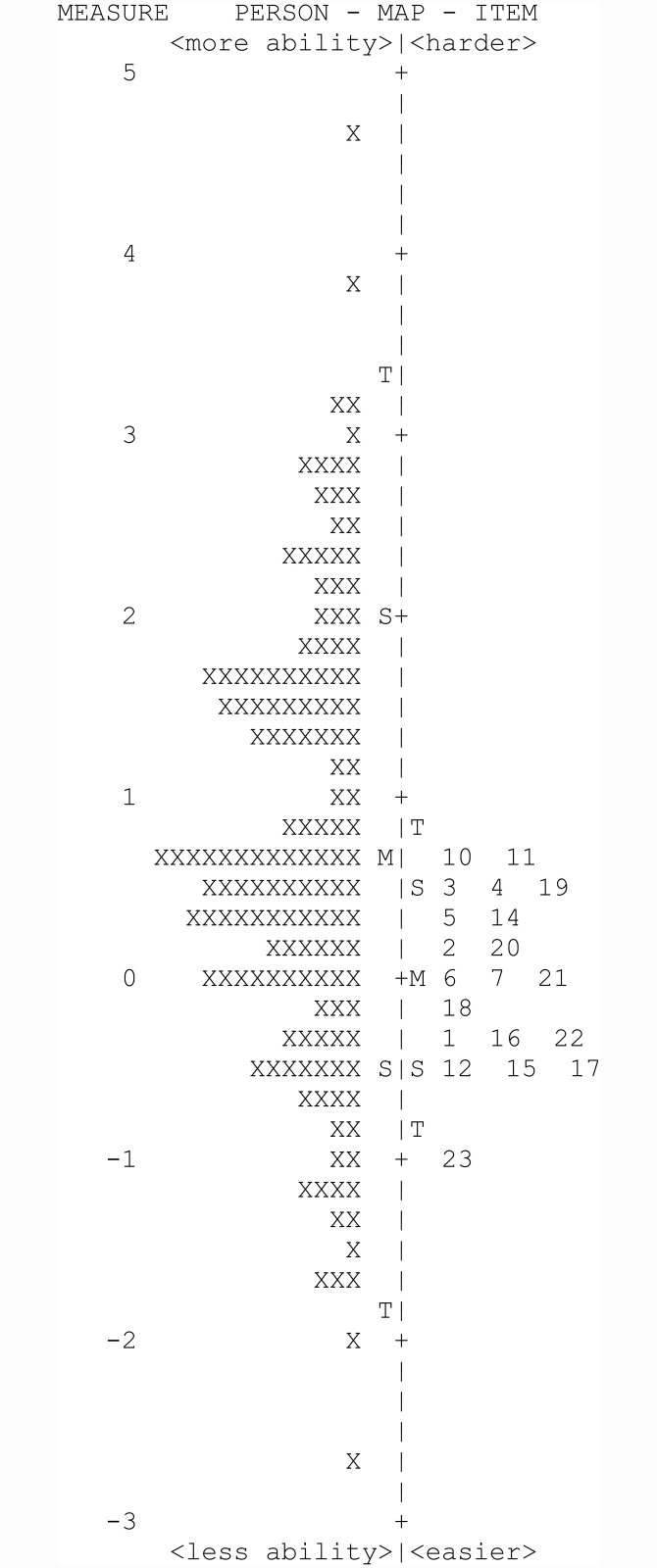
Person-item map of the 20 item emotional health scale.

The remaining 20 items are shown in Table 3 and showed ordered category functions (Andrich thresholds none, -2.48, -0.20, 0.66 and 2.02), each of which was the most probable response at some point on the scale. Person separation was 3.18 and reliability 0.91, and item separation was 3.52 and reliability 0.93, indicating an adequately functioning scale. Adequate targeting of +0.74±1.26 logits was also demonstrated. An item map is shown in Fig 1.

**Table 3:** Characteristics of the questions retained in the ‘overview’ scale.

Item no	Task	Measure	SE	Infit	Outfit	Applicability
10	Deal with misunderstandings from others because of your visual impairment	0.61	0.11	1.11	1.14	134
11	Explain to others what you can and cannot see	0.59	0.11	1.34	1.38	134
3	Deal with frustration, anger or despair	0.53	0.11	0.73	0.73	135
19	Perform your daily activities without suffering from discomfort in the eyes (e g eye strain)	0.46	0.12	0.84	0.85	117
4	Deal with feelings of anxiety	0.42	0.11	0.64	0.64	133
5	Deal with stress	0.42	0.11	0.67	0.72	134
14	Deal with changing roles and relationships because of your visual impairment (with people close to you)	0.33	0.11	0.83	0.80	127
20	Perform daily activities without suffering from other symptoms (such as neck, back or headache)	0.14	0.11	1.32	1.31	121
2	Deal with gloomy or sad feelings	0.11	0.11	0.89	0.87	132
7	Enjoy shared / group activities	0.06	0.11	1.17	1.12	134
6	Deal with feelings of inferiority	-0.02	0.11	1.21	1.11	131
21	Balance your energy during the day (e g so that you have some energy left at the end of the day)	-0.03	0.11	1.10	1.11	120
18	Stay focused and concentrated	-0.23	0.12	0.60	0.60	120
22	Do things in your spare time (such as hobbies or social contacts)	-0.26	0.12	1.16	1.08	118
16	Finish your daily activities in time	-0.30	0.12	1.23	1.24	117
1	Deal with feelings of loneliness	-0.35	0.11	0.82	0.85	127
15	Sustain your daily activities during the day, such as shopping, cooking or arranging things	-0.47	0.12	0.84	0.90	120
12	Ask for help from people you know	-0.48	0.11	1.29	1.20	133
17	Get somewhere without getting too tired	-0.55	0.12	0.86	0.85	120
23	Keep a day and night rhythm	-1.00	0.13	1.34	1.19	116

Item difficulties of tasks retained after preliminary Rasch analysis, in order of the relative difficulty of tasks (most difficult first), also specifying the fit of the item (infit and outfit meansquare) and the number of respondents (maximum 166) to whom the item was applicable.

Examining the differential item functioning (DIF) of the items in the scale, none had noticeable DIF when considering visual impairment registration status or gender. With respect to the use of mobility aids, items 10 (DIF contrast +0.55±0.22 logits) 11 (+0.82±0.21) and 12 (+0.69±0.22) were more difficult for those who do not use mobility aids, and item 15 (-0.74±0.25) was more difficult for those who do use mobility aids. In terms of participants’ age, items 11 (+0.66±0.22) and 12 (+0.66±0.23) were more difficult for those younger than the median age (51 years), and items 15 (-0.66±0.24) and 17 (-0.55±0.24) were more difficult for those aged over 51 years. Item 12 (+0.59±0.22) was more difficult for those who had been visually impaired for greater than the median period (16 years).

Rasch PCA of residuals indicated that the unidimensionality of the overview scale was not good however. The raw variance explained by the measures was 51.2%, lower than the 60% minimum suggested. Additionally, the raw variance explained by the items in the principal Rasch analysis (7.8%) was less than the unexplained variance in the first contrast (8.4%), indicating the presence of a significant second dimension. There were 2 contrasts with eigenvalues greater than 2 units. The first (3.4 eigenunits) included 5 items (3, 2, 4, 5 and 6) with loadings of more than 0.40 onto the contrast. These items relate to dealing with feelings about vision loss. The second contrast (2.9 eigenunits) included 3 items (10, 11, 12) relating to making other people aware of a person’s vision loss.

Identification of contrasts in the data is consistent with the classical factor analysis of Bruijning [29] which resulted in the tasks being separated into the two emotional health goals. Therefore, Rasch analysis was repeated for the tasks underlying the emotional health goal (tasks 1–14) and those underlying the fatigue goal (tasks 15–23) separately, to try to determine appropriate unidimensional subscales.

For the emotional health questions (items 1–14), item reduction was firstly undertaken iteratively until all infit / outfit mnsq values were within 0.6–1.4. The worst fitting item in the initial iteration had a fit of 1.86. Only four items (2, 3, 4 and 5) relating to dealing with feelings resulting from vision loss remained in the subscale (Table 4). Category functions were ordered (Andrich thresholds none, -7.27, -1.26, 2.43 and 6.11), person separation was 2.74 and reliability 0.88, item separation was 2.37 and reliability 0.85, and targeting was +0.88±4.0 logits. The variance explained by the Rasch measures was 77.3%, and the first contrast had an eigenvalue of 2.0.

**Table 4:** Items retained in the 3 sub-scales; a) ‘Feelings’, b) ‘Explaining vision loss’ and c) ‘Fatigue’.

Subscale	Item no	Task	Measure	SE	Infit	Outfit
Feelings	3	Deal with frustration, anger or despair	0.48	0.19	0.94	0.94
	4	Deal with feelings of anxiety	0.17	0.19	0.99	0.94
	5	Deal with stress	0.14	0.19	1.04	1.06
	2	Deal with gloomy or sad feelings	-0.80	0.18	0.95	0.92
Explaining	10	Deal with misunderstandings from others because of your visual impairment	0.81	0.13	1.17	1.16
	11	Explain to others what you can and cannot see	0.72	0.13	0.97	0.98
	8	Be open about your visual impairment with strangers	0.14	0.13	0.96	0.98
	9	Be open about your visual impairment with acquaintances	-0.81	0.13	0.84	0.80
	12	Ask for help from people you know	-0.86	0.14	0.97	1.12
Fatigue	19	Perform your daily activities without suffering from discomfort in the eyes (e.g. eye strain)	0.79	0.13	0.96	0.99
	20	Perform daily activities without suffering from other symptoms (such as neck, back or headache)	0.38	0.13	1.31	1.24
	21	Balance your energy during the day (e.g. so that you have some energy left at the end of the day)	0.16	0.13	0.90	0.85
	18	Stay focused and concentrated	-0.10	0.13	0.68	0.70
	22	Do things in your spare time (such as hobbies or social contacts)	-0.15	0.13	1.38	1.30
	16	Finish your daily activities in time	-0.18	0.13	1.03	1.02
	15	Sustain your daily activities during the day, such as shopping, cooking or arranging things	-0.39	0.13	0.76	0.79
	17	Get somewhere without getting too tired	-0.52	0.13	0.94	1.08

Items are presented in order of the relative difficulty of tasks (most difficult first), and the fit of the item (infit and outfit mnsq) is also given.

Since the initial analysis identified a contrast of items 10–12 which were rejected in the first sub-scale, Rasch analysis was then repeated for the remaining tasks of the emotional health goal, excluding items 2–5 (i.e. items 1 and 6–14), to determine if a second Rasch-stable subscale was present within these items. The worst fitting item in the initial iteration had a fit of 1.58. After item reduction, five items (8, 9, 10, 11 and 12) relating to communicating vision loss to other people remained in this subscale (Table 4). Category functions were ordered (Andrich thresholds none, -3.52, -0.45, 1.06 and 2.91), person separation was 2.29 and reliability 0.84, item separation was 5.19 and reliability 0.96, and targeting was +0.90±2.23 logits. The variance explained by the Rasch measures was 66.8%, and the first contrast had an eigenvalue of 2.0.

For the questions underlying the fatigue goal (items 15–23), after item reduction 8 items (15–22) were retained with fit values 0.6–1.4 (Table 4). The fit of rejected item 23 was 1.59. Category functions were ordered (Andrich thresholds none, -2.77, -0.70, 0.79 and 2.68), person separation was 2.64 and reliability 0.87, item separation was 2.72 and reliability 0.88, and targeting was +0.88±1.78 logits. The variance explained by the Rasch measures was 62.6%, and the first contrast had an eigenvalue of 2.1 (with items 15, 16, and 17 loading >0.4).

The presence of DIF was examined for each of the three individual subscales derived above, using the same demographic variables as considered for the overview scale. The only item demonstrating significant DIF was item 12 within the ‘Explaining’ subscale which was easier (+0.80±0.27 logits) for those younger than the median age.

The emotional health tasks could therefore be considered as: 1) an overview of difficulty with emotional health (Table 3) which is not strictly unidimensional; 2) three specific subscales of questions about feelings, communicating vision loss, and fatigue (Table 4), with good unidimensionality but two of the subscales (‘Feelings’ and ‘Fatigue’) having sub-optimal item separation (<3). With the proviso that neither analysis is perfect in the Rasch sense, the findings are sufficiently robust to be able to say something useful about the emotional health difficulties and needs of people with RP, which are now considered.

### Analysis of Person Measures

Person measures were derived for the emotional health scale and the three subscales outlined above, in order to examine factors affecting responses. Correlations between the different scales were all significant (p = .000 in all cases) but varied in strength, with the overview score relating well to the subscales (Feelings: r = 0.83; Explaining: r = 0.63; Fatigue: r = 0.88), and the correlation between the subscales less strong (Feelings and Explaining: r = 0.41; Feelings and Fatigue: r = 0.56; Explaining and Fatigue: r = 0.31).

To explore the relationship between person measures for each scale and the continuous demographic variables assessed, correlation coefficients were examined. There was no relationship between any of the scales and either duration of visual impairment or age of the participant (Pearson correlation, p>0.05 in all cases).

Person measures for those with different visual impairment registration status were compared using a one way ANOVA. Table 5 indicates there was no significant difference between the registration groups on any of the scales.

**Table 5:** Differences in person measures between participants not registered, registered SI and registered SSI.

	Number	Mean	SD	F	df	p
Overview	No: 14	1.01	0.98	1.37	2, 146	0.26
	SI: 57	0.89	1.31			
	SSI: 78	0.58	1.27			
Feelings	No: 13	1.75	2.81	1.10	2, 132	0.34
	SI: 51	1.31	4.16			
	SSI: 71	0.40	4.07			
Explaining	No: 13	0.55	2.19	2.63	2, 132	0.08
	SI: 51	0.41	2.08			
	SSI: 71	1.31	2.31			
Fatigue	No: 10	1.79	1.44	2.60	2, 119	0.08
	SI: 42	1.12	1.65			
	SSI: 70	0.60	1.87			

For dichotomous variables, person measures were compared using independent sample t-tests. There was a significant difference in person measure dependent on gender across all scales (Table 6), although the significance of the difference in the ‘explaining’ subscale was only marginal. The direction of the difference could be interpreted either as males expressing more ability or as females expressing more difficulty in each case.

**Table 6:** Differences in person measures between male (M) and female (F) participants.

	Number	Mean	SD	t	df	p
Overview	M:62	1.20	1.01	3.95	147	.000
	F: 87	0.41	1.32			
Feelings	M: 59	1.95	3.70	2.81	133	.006
	F: 76	0.04	4.07			
Explaining	M: 59	1.34	2.19	2.03	133	.044
	F: 76	0.55	2.24			
Fatigue	M: 44	1.71	1.33	4.09	120	.000
	F: 78	0.41	1.85			

There was a significant difference in person measure across all scales apart from ‘explaining’ when comparing those who use mobility aids (cane or dog) with those who do not (Table 7). Those who do not use mobility aids expressed more ability with the emotional health items in each case.

**Table 7:** Differences in person measures between those who do not (N) and those who do (Y) use mobility aids (cane and / or guide dog).

	Number	Mean	SD	t	df	p
Overview	N: 74	1.15	1.11	4.15	147	.000
	Y: 75	0.33	1.27			
Feelings	N: 71	2.20	3.33	4.30	133	.000
	Y: 64	-0.59	4.20			
Explaining	N: 71	0.86	2.40	-0.22	133	.828
	Y: 64	0.94	2.06			
Fatigue	N: 51	1.58	1.41	3.90	120	.000
	Y: 71	0.37	1.86			

Stepwise multiple regression analyses were carried out for each scale to determine the principal demographic factors associated with person measure scores. Age, duration of visual loss, registration status, gender and use of mobility aids were considered as independent variables (Table 8). The use of mobility aids and female gender were associated with increased difficulty with emotional health in the overview scale and in 2 of the 3 subscales, with younger age also associated with more difficulty explaining visual loss. The proportion of variance explained by these factors was relatively low however (7–22%).

**Table 8:** Stepwise multiple regression models indicating the demographic variables accounting for a significant proportion of the variance in person measures for each of the emotional health scales examined.

	Factor	R^2^	B	SE (B)	F	df	p
Overview	Mobility aids	0.11	-0.75	0.19			
	Gender	+0.08	-0.72	0.19	16.42	2, 145	.000
Feelings	Mobility aids	0.12	-2.70	0.64			
	Gender	+0.05	-1.73	0.65	13.42	2, 131	.000
Explaining	Age	0.04	+0.03	0.01			
	Gender	+0.03	-0.77	0.38	5.13	2, 131	.007
Fatigue	Gender	0.12	-1.22	0.31			
	Mobility aids	+0.10	-1.13	0.30	16.54	2, 118	.000

The R^2^ value represents the proportion of variance in the data explained by the model parameter, with the increase in variance explained given for subsequent factors to the first selected. B represents the regression coefficient of the predictor variable, and the standard error associated with this value is also given.

## Discussion

The purpose of this study was to understand the impact of RP on emotional health. In order to do this, evaluation of the instrument used to determine emotional health difficulties was first necessary. Applying Rasch analysis to the emotional health task questions of the D-AI allows an evaluation of the performance of the instrument, and provides information about the difficulties experienced by this group. Items were relevant to a high proportion of the people with RP assessed in this study (≥70%). Considering all the tasks together, after removal of three poorly fitting items (refusing help from people you know, and being open about your visual impairment with strangers and acquaintances) the scale performed well, with adequate category functioning, item fits, person and item separation and targeting. The unidimensionality of the scale was not ideal, with two contrasts identified, suggesting that the scale might be usefully broken down into subscales. However, the overall scale is still of use in order to compare the relative difficulty of different items.

Breaking down the emotional health tasks into subscales produced three sets of items relating to unidimensional constructs of ‘feelings’, ‘explaining vision loss’ and ‘fatigue’. Of note is that these very well reflect the original D-AI task structure of ‘Handle feelings’, ‘Acceptance’ and ‘Feeling Fit’ [24]. The feelings and acceptance tasks were subsequently merged into a single ‘emotional health’ goal on the basis of classical factor analysis [25, 29]. The present analysis suggests that keeping these two sections separate may have benefits in reflecting slightly different constructs.

Each of the three subscales of the present analysis behaves well in Rasch analysis, although two of the scales (‘Feelings’ and ‘Fatigue’) have item separations (2.37 and 2.72 respectively) that are slightly lower than the optimal value of 3 [36]. Item separation is used to indicate how well the item hierarchy is defined in the scale, and values less than 3 indicate that the ordering of item difficulties may not be precise. Low item separation can be seen either if the number of people sampled is too small to accurately locate the item difficulties, or if the items have a relatively narrow range of difficulties [36]. The latter is the more obvious problem here: by narrowing the range of items in each subscale to improve unidimensionality, variation in item difficulty is then compromised.

Whilst Rasch analysis allows retention of items that conform to a unidimensional construct, and rejects items that are not responded to similarly to others, there is a danger that the strictness of the Rasch model eliminates useful information. In this study, items were retained with fits of 0.6–1.4, in keeping with previous literature [4, 35], which resulted in rejection of a quarter of the items in the subscales (6 out of 23). Others have advocated even stricter limits of 0.7–1.3 [20]. However, it has been suggested that items with fits of 0.5–15 provide useful information, and that retaining items with fits of up to 2.0 (which would include all the items rejected in the present analysis) does not damage the integrity of the scale [35]. It is worth considering whether such strict observance of fit criteria is necessary for instruments assessing rehabilitation needs.

Whilst neither the overview nor the three subscales respond perfectly to Rasch analysis, either approach might be considered by future researchers utilising this section of the D-AI, depending on the nature of the question being posed and the relative importance of strict unidimensionality, strict location of item difficulties or provision of a comprehensive overview of difficulties. The overview scale considers the greatest range of potential rehabilitation needs and gives the most useful comparison of how difficult items are, but the three subscales are perhaps more useful for considering how well the questionnaire behaves. However, by examining responses to both the overall scale and the subscales, themes emerge that can help understanding of the emotional health needs of those with RP so as to inform the requirements of rehabilitative interventions.

In developing the D-AI through focus groups and psychometric analyses, Bruijning and colleagues [21, 24] have identified appropriate and relevant areas to consider in terms of emotional health, but which of these are the most difficult areas has not previously been evaluated. To examine the most challenging emotional health tasks for those with RP, the item difficulties of the Rasch analysed overview scale can be examined (Table 3). The most difficult tasks relate to communicating visual loss to other people: dealing with misunderstandings from others because of visual impairment, and explaining to others what you can and cannot see. Addressing these difficulties could require training for the person with RP in how to express their visual loss to others. Equally, a better understanding of different types of visual loss and their effects by the general public would be helpful in addressing this issue. Training courses such as ‘My Guide’ in the UK [41], which introduces basic sighted guiding techniques and an understanding of how visual loss can affect people, can only be helpful in raising awareness of visual loss to all.

The fourth most difficult task was ‘performing daily activities without suffering discomfort in the eyes, such as eyestrain’. Utilising residual vision that does not provide a comfortable level of vision for required tasks will be tiring. Possibly this finding highlights the importance of regular eye examinations or low vision assessments to ensure that refractive corrections, tints, lighting and low vision aids provide as much functional reserve as possible between visual function and required tasks.

The other most difficult emotional health tasks involved dealing with feelings of frustration and anger, stress and anxiety. Dealing with such feelings is often one of the topics addressed in self-management programs based on problem-solving approaches, which have been shown to improve function and reduce emotional distress in older adults with AMD [42]. There is also some evidence that the effects of such programs are greater for those whose emotional health is poorer initially [43, 44]. However, the efficacy of self-management programs for people of younger age or other causes of vision loss is not yet known [42], and there is some suggestion that programs may be less effective with heterogeneous groups [19].

One study that has evaluated a self-management program in a group that included participants with RP [44] found that participants achieved lasting improvements specifically in dealing with lonely and sad feelings. However, such aspects are not found to be particularly difficult by the people with RP in the present study: ‘dealing with sad feelings’ was the ninth most difficult item of 20 in the overview scale (+0.11 logits) and ‘dealing with feelings of loneliness’ was sixteenth most difficult (-0.35 logits). The most difficult aspect for people taking part in the self-management program was feeling frustrated or annoyed by their eyesight, consistent with the finding in the present study of ‘dealing with frustration, anger or despair’ being the third most difficult item (+0.52 logits). Unfortunately, whilst peer support improved this aspect of quality of life in the short term, the beneficial effects had disappeared after 6 months [44]. Different approaches to provision of emotional support may be needed to target the areas that people with RP find most difficult in terms of maintaining their emotional health.

In this study, some demographic factors were found to be associated with greater difficulty with emotional health tasks. The use of mobility aids was found to be associated with greater difficulty in all the scales apart from ‘explaining visual loss’ (Tables 7 and 8). A mobility aid not only improves the ability for safe travel, but also acts as an indicator of visual impairment for the user, perhaps explaining why mobility aid users did not find greater difficulty than non-users in their ability to explain their visual loss to others. For the other scales, it is perhaps surprising that those using mobility aids express greater difficulty with emotional health tasks, as it might be considered that accepting a mobility aid could indicate better acceptance of visual loss [45] and better emotional status. However, the finding of increased difficulty in emotional health with the use of mobility aids might be suggested to reflect that those using mobility aids are having greater functional difficulty in general, since users are older, with a longer duration of visual impairment, and more severe impairment by registration status. None of these factors related directly to emotional health scores in this sample, but previous research has shown that both depression and anxiety increase as visual function deteriorates in RP [46], and that depression can be mediated by functional disability [47].

Female gender was also associated with greater difficulty in all scales, particularly the ‘fatigue’ subscale. It is not clear from this association whether women find greater difficulty with the emotional health aspects of sight loss in RP, or whether it may be more socially acceptable for women to express such difficulty with emotional issues than men. It is known however that women have a greater prevalence of depressive disorders [48]. The only significant difference in the characteristics of the men and women in this sample were that men reported a longer duration of visual impairment than the women, but this factor was unrelated to emotional difficulties itself.

However, it should be noted that whilst statistically significant, the amount of variance explained by these predictors in each multiple regression model was low (maximum of 20%). Other factors known to be associated with better psychosocial outcomes with vision loss, such as depression, acceptance of visual loss, and social support [16, 17] could be better at targeting people most likely to be experiencing difficulty with emotional health and needing support, and it is a limitation of the present study that these were not assessed in detail. Difficulties with the D-AI emotional health goals and task scales have been shown to relate to both depressive symptoms and adaptation to visual loss in cross-sectional analysis [29], and longitudinally are associated with general health status [29].

The demographic factors of age, duration of vision loss, and severity of visual loss as given by visual impairment registration status were not associated with difficulties with emotional health tasks. In a cross-sectional sense, it does not seem that emotional difficulties become easier or more difficult over time or with severity of visual loss. This is consistent with previous findings in a group with mixed causes of visual impairment [49], but contrary to the findings of others [17] who have found severity of vision loss does relate to adaptation to visual loss. The present cross-sectional study does not tell us about the change in difficulty of emotional health tasks for an individual over time, although difficulties in the three subscales have been found to remain stable over the period of a year in people with mixed causes of visual impairment, even when undergoing rehabilitation [29]. However, the present findings do suggest that difficulties with emotional health and the potential need for emotional support in RP can be substantial at any time following diagnosis.

People with RP may have different emotional health priorities to people with other causes of visual impairment. In general, RP has a younger age of presentation than many other visually debilitating conditions: many people with RP will be working or have a lifetime of work ahead of them, as opposed to other causes of visual impairment such as macular degeneration where this is less likely to be an issue. RP also generally has a progressive nature rather than a sudden onset. In terms of the emotional consequences of vision loss, the progressive nature might be an advantage as people can come to terms with vision changes gradually, or may be a disadvantage as continued adjustment to continually changing vision may be necessary and concern about prognosis higher. It therefore cannot be assumed that the findings presented here would be applicable to people with visual impairment due to other causes. Previous work has suggested that people with RP use various strategies to help them manage the stress of progressive vision loss, including taking actions to maintain independence and keeping vision loss in perspective [50], and the use of complementary and alternative therapies [51]. Even so, adjustment to visual loss in RP has been suggested to differ from that experienced by people with diabetes, another progressive condition with potential associated visual loss [52].

In summary, the task questions of the emotional domain of the D-AI were Rasch analysed and seen to be useful in assessing the emotional health of people with RP. The most difficult emotional health tasks for people with RP relate to communicating visual loss to other people, dealing with negative feelings, and performing daily tasks without suffering ocular discomfort. Rehabilitation interventions that target these areas of greatest difficulty are needed. Greater difficulty was associated with the use of mobility aids and female gender, and difficulty was not associated with age, or duration or severity of visual loss.
